# Gallic acid showed neuroprotection against endoplasmic reticulum stress in rats

**DOI:** 10.1590/acb400925

**Published:** 2025-01-13

**Authors:** Abdulmutalip Karaaslanlı, Mehmet Cudi Tuncer, Fırat Aşır, Tuğcan Korak

**Affiliations:** 1 Van Yüzüncü Yıl University – Department of Brain and Neurosurgery – Van – Turkey.; 2 Dicle University – Faculty of Medicine – Department of Anatomy – Diyarbakir – Turkey.; 3 Dicle University – Department of Histology and Embryology – Medical Faculty – Diyarbakır – Turkey.; 4 Kocaeli University – Department of Medical Biology – Medical Faculty – Kocaeli – Turkey.

**Keywords:** Gallic Acid, Caspases, Endoplasmic Reticulum Stress, Spinal Cord Injuries

## Abstract

**Purpose:**

We aimed to investigate the role of gallic acid treatment on spinal cord tissues after spinal cord injury (SCI) and its relationship with endoplasmic reticulum (ER) stress by histochemical, immunohistochemical, and in-silico techniques.

**Methods:**

Thirty female Wistar albino rats were divided into three groups: sham, SCI, and SCI+gallic acid. SCI was induced by dropping a 15-g weight onto the exposed T10-T11 spinal cord segment. The SCI+gallic acid group received 25 mg/kg of gallic acid intraperitoneally daily for one week. Histopathological, immunohistochemical, and silico analyses were performed.

**Results:**

Histological analysis revealed improved neural cell survival and tissue integrity in the SCI+gallic acid group compared to the SCI group. Caspase-12 expression was significantly increased in the SCI group, indicating elevated ER stress and apoptosis. Gallic acid treatment resulted in a marked reduction in caspase-12 expression in neurons, neuroglia, and endothelial cells, suggesting decreased ER stress.

**Conclusion:**

Gallic acid exhibits significant neuroprotective effects against ER stress and cellular damage in a rat model of SCI. The in-silico analysis revealed apoptotic and immune-related pathways in which gallic acid showed neuroprotective effects by regulating caspase-12. These results suggest that gallic acid may be a promising therapeutic agent for mitigating secondary damage post-SCI.

## Introduction

Spinal cord injury (SCI) is a complex and devastating condition that affects multiple body functions^1^. In the first 24 hours after SCI, microglia, the specialized macrophages of spinal cord tissue, becomes activated. This activation leads to the production of free oxygen radicals, mitochondrial dysfunction, and the leakage of potassium and genetic material from damaged cells^1^^,^ 2. The primary causes of SCI include flexion, extension, dislocation, distraction, fractures, ruptured disc material, gunshot wounds, contusions, lacerations, and compression. Secondary injuries occur in hours after the primary injury due to metabolic and biochemical factors^3^^,^ 4. These secondary injuries initiate a cascade of events leading to endogenous cell death. Ischemia-induced energy deficiency is a hallmark of secondary SCI. As a cellular response to hypoxia, cells swell and intracellular Ca2+ ion levels increase, activating inflammatory and apoptotic pathways^2^^,^ 4 ^,^ 5.

Gallic acid, a trihydroxybenzoic acid, is found in various herbal medicines, foods, and beverages6. Numerous studies have demonstrated its potential anticancer activities in vivo and *in vitro* 6 ^,^ 7. Beyond its antitumor properties, gallic acid exhibits antioxidative, anti-inflammatory, antidiabetic, antihypercholesterolemic, cardioprotective, antifibrotic, and antihypertensive effects^6^^,^ 8 ^,^ 9. Looking at the relationship between gallic acid and interleukin, interleukin-12 (IL-12) plays a pivotal role not only in immune responses, but also in cellular processes such as endoplasmic reticulum (ER) stress-induced apoptosis and the response to various apoptotic stimuli. IL-12, a key pro-inflammatory cytokine, is primarily involved in activating immune cells and promoting the Th1 immune response. However, recent studies have revealed that IL-12 is also implicated in modulating cellular stress mechanisms, including apoptosis triggered by endoplasmic reticulum stress.

ER stress occurs when the accumulation of misfolded proteins overwhelms the protein-folding capacity of the ER, leading to the activation of the unfolded protein response (UPR). The UPR attempts to restore normal function, but, if the stress persists, it triggers apoptotic pathways. IL-12 has been found to influence this process by modulating the immune and inflammatory responses that can either exacerbate or alleviate ER stress-induced apoptosis. Additionally, IL-12 contributes to the cellular response to various apoptotic stimuli, impacting the balance between survival and cell death in different contexts^10^^–^ 13.

For this reason, given this involvement of IL-12 in apoptotic processes, therapeutic agents like gallic acid, which has demonstrated antioxidant, anti-inflammatory, and anticarcinogenic effects, may be of great significance. Research shows that the administration of gallic acid reduces IL-12 expression, which in turn may decrease ER stress-induced apoptosis and modulate the response to other apoptotic stimuli. This highlights the potential of gallic acid in conditions in which both inflammation and apoptosis are key pathological factors.

The endoplasmic reticulum is crucial for protein folding and sorting^14^. Cellular stress conditions such as glucose deprivation, depletion of ER calcium stores, free radical exposure, and the accumulation of unfolded or misfolded proteins disrupt ER function, triggering the unfolded protein response^14^^,^ 15. The precise molecular mechanisms by which ER stress leads to neuronal survival or death remain unclear, particularly in the context of spinal cord injury^16^.

ER stress-induced neuronal apoptosis is a key pathological process in SCI^14^. Studies have shown increased expression of C/EBP homologous protein (CHOP) and Bax, as well as caspases 3-9 in neurons associated with ER stress and apoptosis following SCI^17^. In an experimental SCI model by Penas et al.^18^, ER stress was induced by an unbalanced UPR, with increased CHOP expression and apoptotic activity. Heat shock proteins (HSPs), particularly HSP70, are important modulators of SCI. HSP70 expression decreases in experimental SCI models^19^^,^ 20. HSP70 prevents apoptosis by inhibiting the activation of procaspase-9 by Apaf-1, thus blocking its conversion to caspase-3^21^. Springer et al. reported that, in early experimental SCI, procaspase-9 is activated by Apaf-1 in the trauma area, leading to its conversion to caspase-3 and subsequent neuronal apoptosis^22^. Hypoxia-inducible factor-1α (HIF-1α) expression increases in SCI and plays a protective role post-injury^23^.

The aims of this study were to elucidate the molecular mechanisms of secondary damage following SCI and to evaluate the potential therapeutic role of gallic acid in mitigating this damage. In addition to examining the effects of free oxygen radicals, mitochondrial dysfunction, and cellular stress induced by SCI, this study focused on the impact of ER stress on neuronal survival and death. The study aimed to explore the anti-inflammatory and free radical scavenging properties of gallic acid in reducing secondary damage due to SCI. This research sought to significantly advance the understanding of cellular and molecular changes post-SCI and to determine the potential protective effects of gallic acid by immunohistochemical, ultrastructural and in-silico analysis techniques.

## Methods

### Experimental design

The ethical permission was obtained from Animal Experimentation Local Ethics Committee of Animal Experiment, Dicle University (approval date: 06.19.2024, approval no.: 2024/15). In this study, 30 female Wistar albino rats were used. The experimental animals were divided into three groups of 10 rats each. They were fed with unlimited access to food and water, and maintained in a controlled environment with a 12-hour light/dark cycle (from 8 a.m. to 8 p.m.) at the temperature of 23 ± 2°C. Gallic acid (catalog no.: 842649, Merck, Germany) was commercially purchased for use in the experimental procedure.

### Surgical operation

- Sham group: After anesthesia, all rats were fixed on the operating table, in the prone position, the skin of the thoracic region was opened with a midline incision, the paravertebral muscles were dissected, and the T10-T11 laminae were exposed. No other procedures were performed, and the animals’ back skin were sutured. After the experiment, 1 cc of physiological saline was given to the animals intravenously (i.v.) once a day for one week;
- SCI group: After anesthesia, all rats were fixed on the operating table, in the prone position, the skin of the thoracic region was opened with a midline incision, the paravertebral muscles were dissected, and the T10-T11 laminae were exposed. After T10-T11, laminectomy was performed and spinal cord exposure was achieved, the animals were placed in the trauma apparatus. A 10-cm long cylindrical tube was fixed to the area in which laminectomy was performed. Spinal damage was created by dropping a metal weight weighing 15 g down the tube. Immediately after the trauma, the muscle and skin incision were sutured. After the experiment, 1 cc of physiological saline was given to the animals i.v. once a day for one week;
- SCI+gallic acid group: Animals before the experiment. After anesthesia, all rats were fixed on the operating table, in the prone position, the skin of the thoracic region was opened with a midline incision, the paravertebral muscles were dissected, and the T10-T11 laminae were exposed. After T10-T11 laminectomy was performed and spinal cord exposure was achieved, the animals were placed in the trauma apparatus. A 10-cm long cylindrical tube was fixed to the area in which laminectomy was performed. Spinal damage was created by dropping a metal weight weighing 15 g down the tube. Immediately after the trauma, the muscles and skin incision were sutured. After the experiment, the animals were given 25 mg/kg gallic acid i.v. once a day for one week^24^.

After the experimental protocol was completed (at the end of the seventh day), the experimental animals were sacrificed by exsanguination under general anesthesia. A piece of the dissected spinal cord tissues was subjected to electron microscopy analysis, and the remaining part was subjected to routine histological tissue preparation.

### Histopathological examination

The rats were euthanized by under general anesthesia and sacrificed. Spinal cord tissues were fixed in formaldehyde solution, dehydrated in a graded alcohol series (80, 90, 96% ethanol), and then embedded in paraffin blocks. To identify histopathological changes in spinal cord, the tissues sections were cut from paraffin blocks, dewaxed and stained with hematoxylin-Eosin. Sections were cleared in xylene and mounted and examined under a Zeiss Imager Axio A2 photomicroscope.

### Immunostaining

Immunohistochemistry with caspase 12 (catalog no.: sc21747, Santa Cruz, United States of America, dilution ratio: 1/100) was performed according to the biotin-streptavidin peroxidase complex method described by Ayaz et al.^25^. Initially, tissue samples were dewaxed in xylene and rehydrated in ethyl alcohol. Endogenous peroxidase activity was blocked by treating the samples with 3% H2O2 (catalog no.: TA-015-HP, ThermoFischer, Waltham, MA, United States of America) for 20 minutes. Antigen retrieval was conducted using citrate buffer (pH 6.0) for 10 minutes at 90°C. To block nonspecific proteins, a blocking solution (catalog no.: TA-015-UB, ThermoFischer, Waltham, MA, United States of America) was applied for 8 minutes. Subsequently, the tissue sections were incubated overnight with the diluted primary antibodies, followed by immersion in 1x phosphate-buffered saline (PBS).

The sections were then incubated with a biotinylated secondary antibody (catalog no.: TP-015-BN, ThermoFischer, Waltham, MA, United States of America) for 15 minutes, immersed again in 1x PBS, and incubated with streptavidin peroxidase (catalog no.: TS-015-HR, ThermoFischer, Waltham, MA, United States of America) for another 15 minutes. The sections were then treated with a chromogen solution (diaminobenzidine, catalog no.: TA-001-HCX, ThermoFischer, Waltham, MA, United States of America), stained with Gill III hematoxylin for 1 minute. Sections were cleared in xylene and mounted and examined under a Zeiss Imager Axio A2 photomicroscope.

### Electron microscopy examination

Lead acetate-uranyl citrate staining was applied to the spinal cord sections for electron microscopy. The sections were examined using a Jeol JEM-1010 transmission electron microscope at Dicle University Science and Technology Application and Research Center. Cytopathological changes were photographed using a side-mounted TEM CCD camera (Gatan ES500W Erlangshen).

### In-silico analysis

In-silico analyses were conducted to investigate the potential pathways through which gallic acid reveals a neuroprotective effect by reducing ER stress via the modulation of caspase-12. Protein-protein interaction networks were constructed using Cytoscape software (https://cytoscape.org/, version 3.10.2). For 100 gallic acid targets, the Search Tool for Interactions of Chemicals module was used, while for 300 Caspase-12 protein interactors, the Search Tool for the Retrieval of Interacting Genes/Proteins module was employed. Networks were generated at a medium confidence level (0.400), and common target proteins were identified by intersecting these networks. Subsequently, Reactome pathway annotation was performed using Enrichr (https://maayanlab.cloud/Enrichr/) based on the shared proteins. The top 10 statistically significant pathways (*p* < 0.05) were listed in ascending order of their *p*-values^26^.

## Results

### Histochemical findings

**Figure 1:**
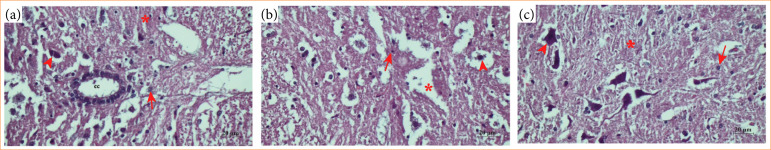
Microscopic sections of spinal cords belonging to **(a)** sham group, **(b)** spinal cord injury group (SCI), and **(c)** SCI+gallic acid group. Hematoxylin eosin staining, scale bar: 20 µm, magnification: 40×.

The histopathological images of spinal cord sections belonging to the groups are shown in Fig. 1. In the microscopic section of the sham group, it was observed that the neurons were smooth, and the neural tissue was regular and well protected. It was observed that the ependymal cells in the central canal were arranged in the form of a high cuboidal epithelium, and the neuroglia cells were prominent (Fig. 1a). In the SCI group, significant cell damage and structural deterioration were observed. Deteriorations and degeneration in neuropil formation were observed. In addition to neuron and neuroglia loss and degeneration, apoptotic nucleus images were present (Fig. 1b). There is a significant improvement in tissue structure in the SCI+gallic acid group compared to the SCI group. With the effect of gallic acid treatment, tissue order was regained to some extent, and neuron and glial cells showed less degeneration and apoptosis. The organization of neuropile was more regular and organized (Fig. 1c). This demonstrates the neuroprotective effects of gallic acid.

Arrow: neuroglial cells; arrowhead: neurons; *neuropile. Source: Elaborated by the authors.

### Immunohistochemical findings

**Figure 2:**
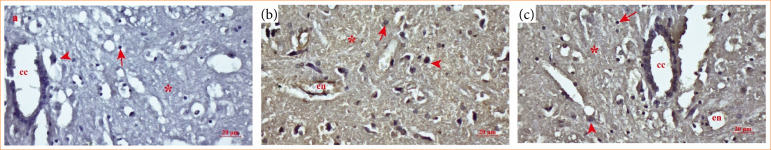
Caspase 12 expression in cross sections of spinal cord tissues belonging to **(a)** sham group, **(b)** spinal cord injury (SCI) group, and **(c)** SCI+gallic acid group. Caspase 12 immune staining, scale bar: 20 µm, magnification: 40×.

To demonstrate ER stress, tissue sections of the spinal cord were stained with caspase-12 immune staining and are shown in Fig. 2. Caspase 12 expression was generally observed as negative in ependymal cells, neuroglial cells and neurons in the central canal (Fig. 2a). After SCI, caspase 12 expression in neuropil areas was higher in the SCI group than in the sham group. Caspase 12 immune reactivity was significantly increased in neurons, neuroglia and vascular endothelial cell nuclei in the spinal cord. This shows that apoptotic action occurs in these cells due to increased ER stress after SCI (Fig. 2b). After gallic acid treatment, caspase 12 immunoexpression in both ependymal cells, neuron and neuroglial cells decreased significantly in the SCI+gallic acid group compared to the SCI group. There was a decrease of caspase 12 expression in endothelial cell in vessels (Fig. 2c). Gallic acid protected the local neural tissue by supporting cell survival, which indicates that it has a neuroprotective effect against ER stress.

Arrow: neuroglia; arrowhead: neurons; *neuropile; cc: central canal; en: endothelial cell. Source: Elaborated by the authors.

### Ultrastructural findings

**Figure 3:**
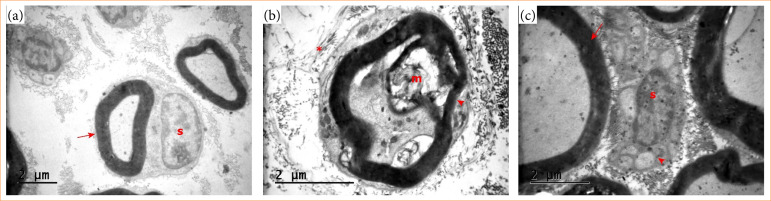
Transmission electron microscopy of spinal cord sections. **(a)** Sham group, normal axonal ultrastructure, arrow: myelin sheath, s: Schwann cell nuclei; **(b)** spinal cord injury (SCI) group, disrupted axon, arrowhead: disorganized myelin sheath with degenerated Schwann cell, m: macrophage, *degenerated extracellular matrix; **(c)** SCI+gallic acid group, improved ultrastructure of axons; arrow: regular myelin sheath, arrowhead: unmyelinated small axons, s: Schwann cell.

Source: Elaborated by the authors.

Ultrastructural images of spinal cord tissues taken with transmission electron microscopy are shown in Fig. 3. In the sections of the sham group, the myelin sheath was seen as a ring, and the Schwann cell nucleus was seen around it. Myelin sheaths were properly placed, and euchromatic and heterochromatic areas were observed in the Schwann cell nucleus (Fig. 3a). In the SCI group, degeneration was observed in the myelin sheath. Macrophage cells phagocytose the myelin sheath, and the structures in the extracellular matrix were degenerated. In the degenerated axon, the organization of the myelin sheath was disrupted. It became thinner, vacuoles were formed, and microglia surrounded it (Fig. 3b). In the SCI+gallic acid group, the myelin sheath organization improved, the Schwann cell membrane was evident, and there were very few axons without myelin sheath (Fig. 3c).

### In-silico analysis findings

**Figure 4:**
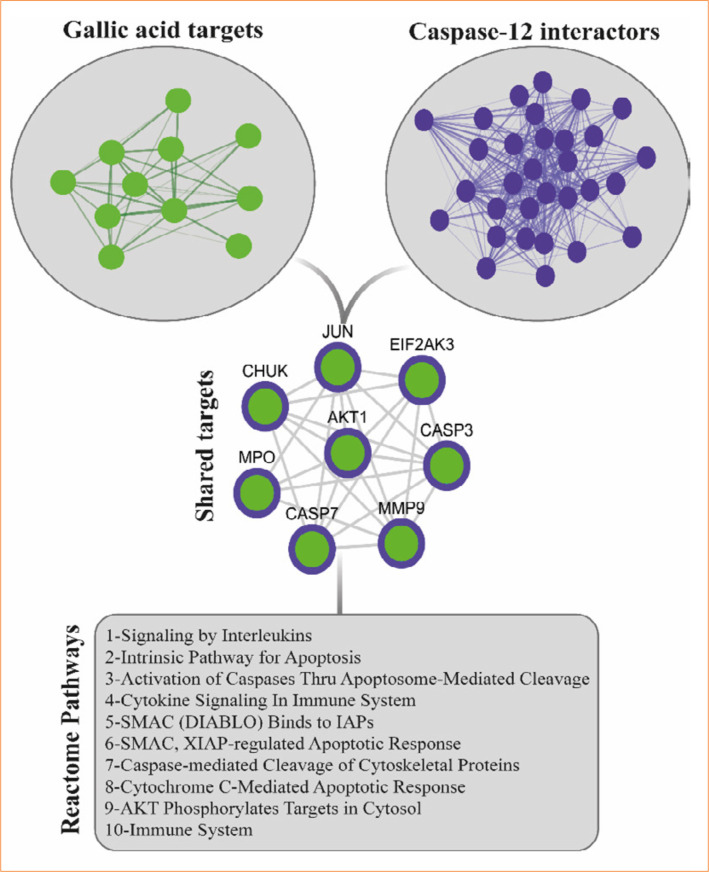
The common interactors of gallic acid and caspase-12, along with their 10 pathway annotations. The Reactome pathway analysis was based on eight common proteins, presenting statistically significant pathways with a *p*-value less than 0.05.

Source: Elaborated by the authors.

The intersection of the protein interactors of gallic acid and caspase-12 resulted in the identification of eight common proteins: AKT serine/threonine kinase 1 (AKT1), caspase-3 (CASP3), CASP7, CHUK, eukaryotic translation initiation factor 2 alpha kinase 3 (EIF2AK3), jun proto-oncogene (JUN), matrix metallopeptidase 9 (MMP9), and myeloperoxidase. Reactome pathway analysis using these proteins yielded 10 significant annotations, as follows: signaling by interleukins, intrinsic pathway for apoptosis, activation of caspases through apoptosome-mediated cleavage, cytokine signaling in the immune system, SMAC (DIABLO) binds to inhibitor of apoptosis protein (IAP), SMAC, X-linked inhibitor of apoptosis (XIAP)-regulated apoptotic response, caspase-mediated cleavage of cytoskeletal proteins, cytochrome C-mediated apoptotic response, AKT phosphorylates targets in the cytosol, and the immune system (Fig. 4).

### Future directions for the neuroprotective role of gallic acid

The findings of this study demonstrate the promising neuroprotective role of gallic acid in mitigating endoplasmic reticulum stress, a critical factor in neurodegenerative diseases. To further explore the therapeutic potential of gallic acid, future studies should focus on evaluating its long-term efficacy and safety in preclinical models of chronic neurodegenerative conditions such as Alzheimer’s disease and Parkinson’s disease. Investigating the molecular mechanisms underlying gallic acid’s modulation of ER stress pathways, including its impact on key mediators such as CHOP, GRP78 (G-protein coupled receptor 78), and protein kinase RNA-like endoplasmic reticulum kinase (PERK), will offer deeper insights into its neuroprotective action. Additionally, clinical trials are warranted to assess the translational value of these findings in human subjects, exploring optimal dosing strategies and potential combinatory effects with existing neuroprotective agents. A broader investigation into gallic acid’s effects on different brain regions and its capacity to enhance synaptic plasticity could open avenues for its therapeutic application in cognitive and memory-related disorders.

## Discussion

In this study, we examined the histopathological changes in spinal cord tissue after SCI and the neuroprotective effects of gallic acid treatment. After gallic acid treatment, histopathology was mostly improved with more neural cells survival and regeneration, suggesting the neuroprotective effects of gallic acid. This histopathological analysis demonstrates the therapeutic effects of gallic acid on SCI. The treatment group exhibited a significant improvement compared to the injury group, and tissue integrity appeared to be better preserved.

SCI causes nervous system injury and defects motor and sensory neurons, leading to oxidative stress and neural tissue damage^27^. Accumulation of reactive oxygen species induces neuroinflammation and apoptosis inducing ER stress in cells^28^^,^ 29. Due to its complex pathophysiology, no exact treatment is available in clinical conditions to significantly improve neurological functions^27^.

Ekinci et al. conducted an experimental SCI model in rats and found that SCI caused an increase of oxidative stress, degeneration of central canal ependymal cells and vascular structures^30^. Similar findings were recorded by Avınca et al.^31^. Biochemically oxidative stress increased, histochemically neuronal degeneration, vascular dilatation, inflammation and apoptotic nuclei were observed. The authors also found that ultrastructural examinations showed loss of membrane and cristae in mitochondria, and dilatation in the endoplasmic reticulum was observed due to degeneration in the neuron structure31. These findings are consistent with our results, suggesting SCI causes neural tissue damage. However, the therapeutic effects of gallic acid on SCI revealed that SCI+gallic acid group exhibited a significant improvement compared to the SCI group, and tissue integrity appeared to be better preserved. Reorganization of myelin sheath, regeneration Schwann cells and increase in myelinated axons indicated protective effects of gallic acid treatment also at ultrastructural level.

Caspases are also involved in SCI pathophysiology and ER stress, for example caspase 3 is activated after traumatic SCI^22^. Activation of caspase 8 and caspase 10 initiates apoptosis after SCI in rodents^32^. Apoptosis causes detrimental neurological damage after SCI^33^. Based on our findings, we can conclude that ER stress was demonstrated in spinal cord tissues using caspase 12 immune staining. After SCI, caspase 12 expression was upregulated in the SCI group compared to the sham group. This indicates that apoptotic action occurs in these cells due to increased ER stress after SCI. Following gallic acid treatment, caspase 12 immunoexpression significantly downregulated in neuronal cells in the SCI+gallic acid group compared to the SCI group. Gallic acid protected the local neural tissue by supporting cell survival, indicating that it has a neuroprotective effect against ER stress.

The in-silico analysis demonstrated critical pathways by which gallic acid reveals its neuroprotective effects through interaction with caspase 12, highlighting potential mechanisms that mitigate ER stress. Pathway analysis of the shared targets of gallic acid and caspase 12 identified significant annotations, including signaling by interleukins, the intrinsic pathway for apoptosis, activation of caspases through apoptosome-mediated cleavage, cytokine signaling in the immune system, and AKT-related pathways. Gallic acid has been shown to be effective against ER stress^34^^,^ 35, a cellular event that occurs due to the persistent accumulation of misfolded proteins, in various studies^14^^,^ 36. It constitutes a fundamental pathogenic mechanism underlying the majority of neurological disorders^37^. Upon failure of the UPR adaptive response, ER stress can activate apoptotic mechanisms through various pathways. While caspases such as 3, 6, 7, 8, and 9 have been implicated in ER stress-induced apoptosis, caspase 12 is considered to play a key role in this process^14^^,^ 36.

Our findings from both in-silico and *in-vivo* studies suggest that caspase-12-associated targets of gallic acid may be involved in apoptotic mechanisms at various stages, reinforcing its potential therapeutic relevance in neuroprotection. On the other hand, the immune system-related pathway annotations indicate that gallic acid may exert neuroprotective effects by modulating inflammatory responses. Research indicates that ER stress directly modulates inflammatory pathways and is closely associated with neuronal death and neuroinflammation^37^^,^ 38. By targeting caspase 12 and associated pathways, gallic acid may attenuate ER stress-induced inflammation, thereby promoting neuroprotection.

This study provides valuable insights into the neuroprotective effects of gallic acid against endoplasmic reticulum stress in rats; however, several limitations should be acknowledged. First, the study was conducted exclusively in an animal model, which may not fully replicate the complexities of human neurodegenerative conditions associated with ER stress. Therefore, the results may not be directly translatable to clinical settings without further investigation. Second, the study focused on a single dose and time point for gallic acid administration, limiting the understanding of dose-response relationships and long-term effects. Additionally, the specific molecular mechanisms through which gallic acid exerts its neuroprotective effects require more detailed exploration, such as through gene expression and pathway analysis. Lastly, other factors such as the influence of different stress pathways or interactions with other neuroprotective agents were not examined, which could further elucidate gallic acid’s therapeutic potential.

## Conclusion

Findings of this study suggest that gallic acid exerts a neuroprotective effect against ER stress and cellular damage after SCI, promotes the survival of neurons and glial cells, and improves tissue regulation. The in-silico analysis unveiled apoptotic and immune-related pathways by which gallic acid exhibits neuroprotective effects through caspase 12 regulation, underscoring potential mechanisms that mitigate ER stress and emphasizing gallic acid’s potential as a neuroprotective agent. Further studies are required to explore the role of gallic acid treatment on SCI and ER stress mechanism.

## Data Availability

All data can be obtained from the corresponding author.
